# Supplementary material for: HIVprotI: an integrated web based platform for prediction and design of HIV proteins inhibitors
Source: J Cheminform. 2018 Mar 9;10:12. doi: 10.1186/s13321-018-0266-y (PMC5845081; doi:10.1186/s13321-018-0266-y)
Supplement: Supplementary file 2 — Additional file 2. Source code of HIVProtI web server. [file 13321_2018_266_MOESM2_ESM.zip › HIVprotI_Source-code/hivprotI/weka-3-6-6/doc/weka/associations/FPGrowth.BinaryItem.html]

FPGrowth.BinaryItem


---


|  |  |  |
| --- | --- | --- |
| **Overview** \| **Package** \| **Class** \| **Tree** \| **Deprecated** \| **Index** \| **Help** \| **Weka's home** | |  |
| **PREV CLASS**   **NEXT CLASS** | **FRAMES**    **NO FRAMES**     **All Classes** |  |
| SUMMARY: NESTED \| FIELD \| CONSTR \| METHOD | DETAIL: FIELD \| CONSTR \| METHOD |  |


---


## weka.associations Class FPGrowth.BinaryItem

```
java.lang.Object
  weka.associations.FPGrowth.BinaryItem
```

**All Implemented Interfaces:**: java.io.Serializable, java.lang.Comparable<FPGrowth.BinaryItem>

**Enclosing class:**: FPGrowth

---

``` public static class FPGrowth.BinaryItem extends java.lang.Object implements java.io.Serializable, java.lang.Comparable<FPGrowth.BinaryItem> ```

Inner class that handles a single binary item

**See Also:**: Serialized Form

---

**Constructor Summary**

`FPGrowth.BinaryItem(Attribute att,
int valueIndex)`


| **Method Summary** | |
| --- | --- |
| `int` | `compareTo(FPGrowth.BinaryItem comp)`             Ensures that items will be sorted in descending order of frequency. |
| `void` | `decreaseFrequency()`             Decrement the frequency of this item. |
| `void` | `decreaseFrequency(int f)`             Decrease the frequency of this item. |
| `boolean` | `equals(java.lang.Object compareTo)` |
| `Attribute` | `getAttribute()`             Get the attribute that this item corresponds to. |
| `int` | `getFrequency()`             Get the frequency of this item. |
| `int` | `getValueIndex()`             Get the value index for this item. |
| `int` | `hashCode()` |
| `void` | `increaseFrequency()`             Increment the frequency of this item. |
| `void` | `increaseFrequency(int f)`             Increase the frequency of this item. |
| `java.lang.String` | `toString()`             A string representation of this item. |
| `java.lang.String` | `toString(boolean freq)`             A string representation of this item. |
| `java.lang.String` | `toXML()` |

**Methods inherited from class java.lang.Object**

`getClass, notify, notifyAll, wait, wait, wait`

**Constructor Detail**

### FPGrowth.BinaryItem

```
public FPGrowth.BinaryItem(Attribute att,
                           int valueIndex)
                    throws java.lang.Exception
```

**Throws:**: `java.lang.Exception`


**Method Detail**

### increaseFrequency

```
public void increaseFrequency(int f)
```

:   Increase the frequency of this item.

    :   **Parameters:**: `f` - the amount to increase the frequency by.

---


### decreaseFrequency

```
public void decreaseFrequency(int f)
```

:   Decrease the frequency of this item.

    :   **Parameters:**: `f` - the amount by which to decrease the frequency.

---


### increaseFrequency

```
public void increaseFrequency()
```

:   Increment the frequency of this item.

---


### decreaseFrequency

```
public void decreaseFrequency()
```

:   Decrement the frequency of this item.

---


### getFrequency

```
public int getFrequency()
```

:   Get the frequency of this item.

    :   **Returns:**: the frequency.

---


### getAttribute

```
public Attribute getAttribute()
```

:   Get the attribute that this item corresponds to.

    :   **Returns:**: the corresponding attribute.

---


### getValueIndex

```
public int getValueIndex()
```

:   Get the value index for this item.

    :   **Returns:**: the value index.

---


### toString

```
public java.lang.String toString()
```

:   A string representation of this item.

    :   **Overrides:**: `toString` in class `java.lang.Object`
    :   **Returns:**: a string representation of this item.

---


### toString

```
public java.lang.String toString(boolean freq)
```

:   A string representation of this item.

    :   **Parameters:**: `freq` - true if the frequency should be included. **Returns:**: a string representation of this item.

---


### toXML

```
public java.lang.String toXML()
```

---


### compareTo

```
public int compareTo(FPGrowth.BinaryItem comp)
```

:   Ensures that items will be sorted in descending order of frequency.
    Ties are ordered by attribute name.

    :   **Specified by:**: `compareTo` in interface `java.lang.Comparable<FPGrowth.BinaryItem>`
    :   **Parameters:**: `comp` - the BinaryItem to compare against.

---


### equals

```
public boolean equals(java.lang.Object compareTo)
```

:   **Overrides:**: `equals` in class `java.lang.Object`

---


### hashCode

```
public int hashCode()
```

:   **Overrides:**: `hashCode` in class `java.lang.Object`


---


|  |  |  |
| --- | --- | --- |
| **Overview** \| **Package** \| **Class** \| **Tree** \| **Deprecated** \| **Index** \| **Help** \| **Weka's home** | |  |
| **PREV CLASS**   **NEXT CLASS** | **FRAMES**    **NO FRAMES**     **All Classes** |  |
| SUMMARY: NESTED \| FIELD \| CONSTR \| METHOD | DETAIL: FIELD \| CONSTR \| METHOD |  |


---
